# Acclimating Fever of Liberia

**Published:** 1845-08

**Authors:** 


*Acclimating Fever of Liberia.*—Since the appointment of Dr. J. W. Lugenbeel to the office of Colonial Physician, in Liberia, important facts have been gathered, which are quite new to the profession, and of such value to the interests of African Colonization, that they should be extensively circulated.

"In regard to the influences of the climate on the physical system," says Dr. L. in the July No. of the African Repository, "I may remark that my experience and observations in reference to myself and many others, have confirmed me in the opinion that the climaterial influences are less deleterious to human health than is generally the case in the United States. Every person who emigrates from a temperate climate to this country, must experience some acclimating process, which may or may not be attended with much fever, according to circumstances—to constitutional predisposition, previous habits of life, &c. In some cases the acclimating fever is violent and fatal in its effects ; but in the large majority of cases, it is mild in its form and yields readily to appropriate treatment. Very few persons die during the first attack of fever ; the principal danger is in consequence of relapses, which, in nine cases in twenty, are the results of personal imprudence, and not the effects of the continued injurious influences of the climate. I find that those persons who have resided in the colony one year or more, and who are able to live comfortably, generally enjoy very good health. The principal cases of sickness are among those who are in indigent circumstances, and in whom poverty and indolence are often associated."—*Boston Med. and Surg. Journ.*

